# Supplementary material for: Investigating the influence of physiologically relevant hydrostatic pressure on CHO cell batch culture
Source: Sci Rep. 2021 Jan 8;11:162. doi: 10.1038/s41598-020-80576-8 (PMC7794228; doi:10.1038/s41598-020-80576-8)
Supplement: Supplementary file 1 — Supplementary Information 1. [file 41598_2020_80576_MOESM1_ESM.zip › S1/IgG/Jan17 standard curve/60ugml.pdf]

===

Acq. Operator : SYSTEM Seq. Line : 4
Sample Operator : SYSTEM
Acq. Instrument : HPLC-0039 Location : 4
Injection Date : 1/17/2019 2:05:14 PM Inj : 1
Inj Volume : 100.000 µl
Acq. Method : C:\Users\Public\Documents\ChemStation\1\Data\TK_CHO_IgG 2019-01-17 13-50-44\CHO_IgG_LC.M
Last changed : 12/4/2018 1:38:41 PM by SYSTEM
Analysis Method : C:\Users\Public\Documents\ChemStation\1\Data\TK_CHO_IgG 2019-01-17 13-50-44\CHO_IgG_LC.M (Sequence Method)
Last changed : 1/17/2019 2:21:39 PM by SYSTEM
(modified after loading) (Current integration events modified)

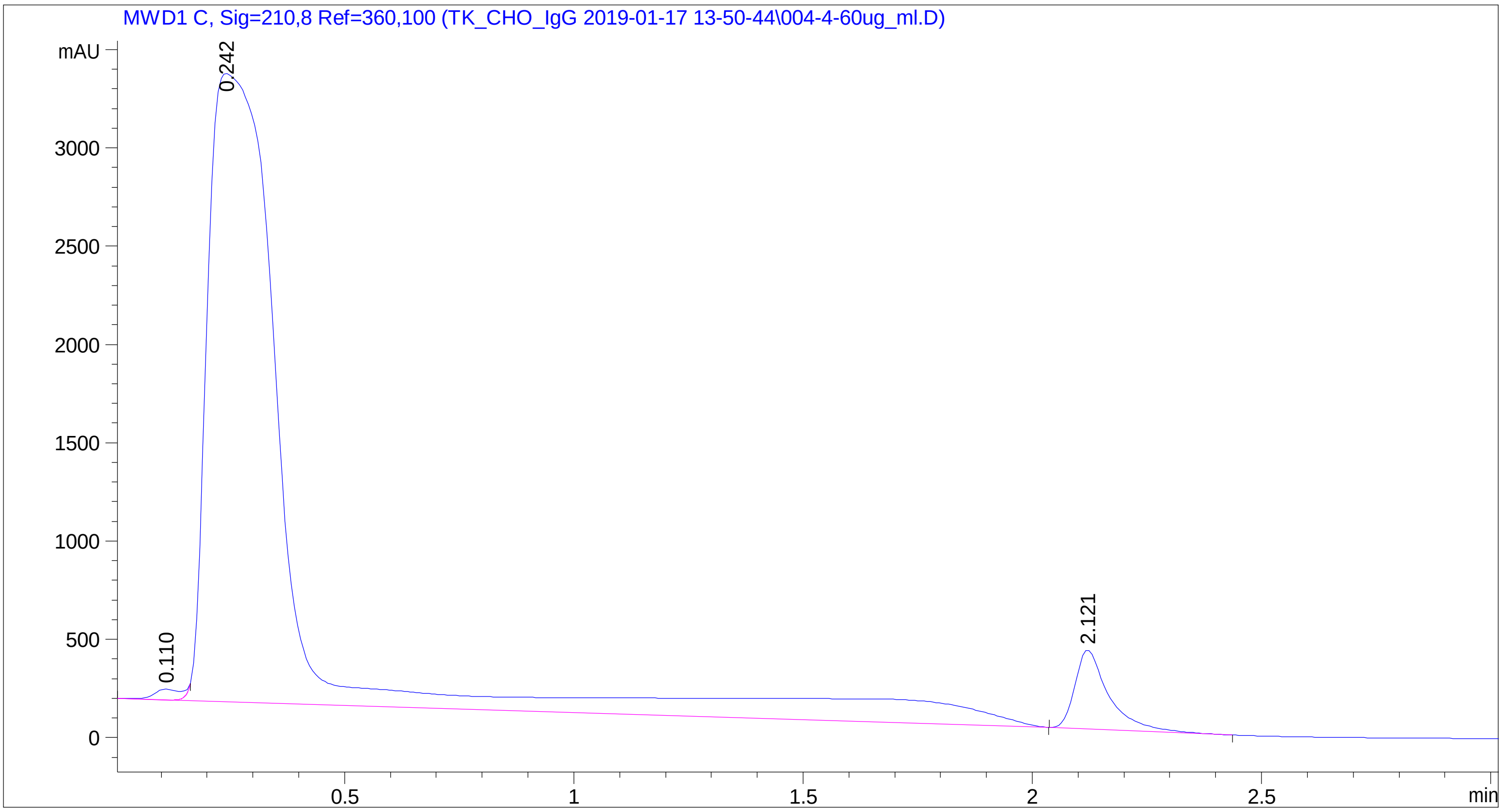

===

Area Percent Report

===

Sorted By : Signal
Multiplier : 1.0000
Dilution : 1.0000
Use Multiplier & Dilution Factor with ISTDs

Signal 1: MWD1 C, Sig=210,8 Ref=360,100

Peak #	RetTime [min]	Type	Width [min]	Area [mAU*s]	Height [mAU]	Area %
1	0.110	BV E	0.0595	226.02879	55.49992	0.5509
2	0.242	VB R	0.1874	3.87376e4	3195.91577	94.4169
3	2.121	BBA	0.0739	2064.62915	402.12674	5.0322

Totals : 4.10283e4 3653.54243

===

*** End of Report ***
